# Application of multi-perspective nursing based on the whole-course ERAS concept during laparoscopic fundoplication in patients with PPI-dependent gastroesophageal reflux disease

**DOI:** 10.4314/ahs.v25i2.21

**Published:** 2025-06

**Authors:** Lina Wang, Ting Zhou, Weiqiong Wang, Wenjie Cai

**Affiliations:** 1 Department of General Surgery, the First Affiliated Hospital of Hainan Medical University, Haikou, China; 2 School of Nursing, Guangdong Medical University, Dongguan, China

**Keywords:** whole-course Enhanced Recovery After Surgery, multi-perspective nursing, proton pump inhibitor -dependent gastroesophageal reflux disease, laparoscopic fundoplication

## Abstract

**Background:**

To examine the impact of a multi-perspective nursing approach using the whole-course Enhanced Recovery After Surgery concept on laparoscopic fundoplicationoutcomes for patients with proton pump inhibitor-dependent gastroesophageal reflux disease.

**Methodology:**

This study was a randomized controlled trial. 98 proton pump inhibitor -dependent gastroesophageal reflux disease patients who underwent aparoscopic fundoplication in our hospital from Jan 2020 to Dec 2022 were randomly divided into two groups using a random number table. Group A (n=49) received multi-perspective nursing based on the gastroesophageal reflux disease concept in addition to routine nursing intervention, while Group B (n=49) received only routine aparoscopic fundoplication perioperative nursing intervention. The groups were compared in terms of first postoperative anal exhaust time, feeding time, time out of bed, hospital stay, Visual Analogue Scale scores before and after surgery, Hamilton Anxiety Scale and Hamilton Depression Scale scores, nursing satisfaction, and complications.

**Results:**

Group A showed significant improvements in various postoperative outcomes compared to Group B, including shorter anal exhaust time, feeding time, time out of bed, and hospital stay (P<0.05). Group A also had lower VAS pain scores, lower Hamilton Anxiety Scale and Hamilton Depression Scale scores, and higher nursing satisfaction scores than Group B (P<0.05).

**Conclusion:**

Multi-perspective nursing with Enhanced Recovery After Surgery can improve gastroesophageal reflux disease patients' recovery, pain relief, and nursing satisfaction post- aparoscopic fundoplication.

## Introduction

Gastroesophageal reflux disease (GERD) is a chronic digestive system disorder. It occurs due to the reflux of stomach contents into the throat, trachea, lungs, or esophagus. These stomach contents are irritating, immunoreactive, and corrosive. As a result of this reflux, individuals experience a series of symptoms and may develop complications. If not effectively treated in a timely manner, it can cause gastritis, precancerous lesions, and even tumors^1^,^2^. Currently, proton pump inhibitors (PPIs) are often used for on-demand long-term treatment of GERD patients, which can achieve good therapeutic effects^3^. However, there are still some PPI-dependent GERD patients with limited efficacy, who need to undergo laparoscopic fundoplication (LF)^4^. Studies have found that targeted care during the perioperative period for GERD patients can strengthen their health education and effectively alleviate pain responses and negative emotions during the perioperative period, promoting postoperative recovery^5^. In the past, conventional LF perioperative care intervention programs were used for GERD patients, but the lack of intervention summaries for postoperative complications and negative patient psychology affected the effectiveness of care interventions^6^. Enhanced Recovery After Surgery (ERAS) is currently the latest innovative concept and treatment strategy internationally, which can promote rapid patient recovery through targeted optimization and improvement of perioperative measures such as preoperative education, intraoperative measures, postoperative rehabilitation treatment, and care, and is widely used in laparoscopic surgery patients^7^. Multidimensional nursing can target interventions from different perspectives based on existing or potential problems of patients to achieve optimal problem-solving nursing intervention methods, which is beneficial to improve the effectiveness of nursing interventions^8^. This study mainly explores and analyzes the application effect of multidimensional nursing based on the whole ERAS concept in PPI-dependent GERD patients undergoing LF.

## Patients and Methods

### Patients

By consulting relevant literature and combining with the statistical principle of n >30, we determined the sample size. We selected 98 patients with PPI-dependent GERD who underwent laparoscopic fundoplication (LF) in our hospital from January 2020 to December 2022. Inclusion criteria were:. 1. Participants must meet the diagnostic criteria for GERD as defined by the Montreal Classification (2018), which includes the presence of troublesome reflux symptoms (such as heartburn or regurgitation) that occur at least twice a week for a minimum duration of 4 weeks.

2. Participants should demonstrate objective evidence of GERD through one or more of the following methods: esophageal pH monitoring showing an abnormal acid exposure time (AET) greater than 6%, or endoscopic findings of erosive esophagitis or Barrett's esophagus.

3. Individuals should have been on PPI therapy for a minimum of 6 months prior to enrollment, with a minimum dosage of once daily.

4. Participants should exhibit typical symptoms of PPI-dependence, including but not limited to rebound acid hypersecretion, symptom recurrence upon PPI discontinuation, and a requirement for escalating PPI doses to achieve symptom control. Exclusion criteria were: concurrent gastrointestinal bleeding or ulcer; concurrent malignant tumors or psychological and mental disorders. Using the random number table method, we divided the patients into Group A and Group B, with 49 patients in each group. Group A had a mean age of (58.95±1.34) years and a disease course of 0.5-8 years with a mean of (2.45±0.30) years, including 23 males and 26 females. The control group had a mean age of (59.01±1.42) years and a disease course of 0.6-7 years with a mean of (2.59±0.31) years, including 19 males and 30 females. There were no significant differences in baseline data between the two groups of PPI-dependent GERD patients who underwent LF (P>0.05), indicating comparability. All protocols of this study have been approved by the Ethics Committee of the First Affiliated Hospital of Hainan Medical University.

## Methods

The group B were implemented routine care interventions during the perioperative period for laparoscopic fundoplication (LF). The methods included: (1) Preoperative care: after patients were admitted to the hospital, they were provided with informational materials such as brochures, graphic and video materials to educate patients and their families about gastroesophageal reflux disease (GERD) and LF-related knowledge. The focus was on explaining perioperative-related precautions, helping patients and their families establish a good understanding of the disease and cooperation for the surgery. Patients and their families were instructed in detail to fast before the surgery, and to assist with bowel preparation and respiratory function exercises. (2) Postoperative care: close attention was paid to wound exudate and gastrointestinal function recovery in patients, and assistance was provided to ensure proper positioning and to develop a postoperative diet plan. (3) Discharge care: Specific postoperative precautions and follow-up appointment times were introduced to patients and their families, and timely medical care was sought if any abnormalities occurred.

The group A was implemented multi-perspective care based on the Enhanced Recovery After Surgery (ERAS) concept on the basis of the group B routine care interventions. The methods included: (1) Establishing a small ERAS multi-perspective care intervention group: a team composed of the attending physician and the operating room nursing supervisor was established to clarify the responsibilities between medical staff and patients, strengthen communication among medical staff, patients and their families, and to develop a scientifically reasonable intervention plan based on the ERAS concept by understanding the patient's basic condition, surgical plan, and personalized nursing needs. (2) Preoperative care: patients were assisted in completing preoperative examination of LF. Educational interventions about GERD and LF-related health knowledge were provided to patients and their families through centralized education and one-on-one communication, to assist patients in preoperative bowel preparation, alleviate anxiety and tension, and to improve treatment and nursing compliance. (3) Preoperatie care before entering the operating room: patients were encouraged through verbal encouragement to adjust their nervous and anxious emotions. (4) Postoperative care: wound exudate and gastrointestinal function recovery were observed during ward rounds, and appropriate pain relief was given if necessary. Assistance was provided to ensure proper positioning and to develop a postoperative diet plan, with a focus on high protein, high fiber, low-fat, and low-sugar diet principles. (5) Rehabilitation training: after patients regained consciousness, they were assisted in cough training and bed-turning and other rehabilitation exercises. Patients were encouraged to get out of bed and gradually increase their activity level based on their recovery status.

### Observation indicators

We compared the postoperative recovery indicators, including the first time of anal exhaust, the first time of oral intake, the first time of getting out of bed, and length of hospital stay, between the two groups of PPI-dependent GERD patients who underwent LF surgery. We evaluated the changes in pain severity using the Visual Analogue Scale (VAS)^11^ at pre-operation, 2 h, 8 h, 12 h, 24 h, and 36 h post-operation. The VAS scale used a 5-point rating system, with higher scores indicating more severe pain reactions. We also assessed negative psychological changes and nursing satisfaction of the two groups before and after intervention using the Hamilton Anxiety Scale (HAMA)^12^, Hamilton Depression Scale (HAMD)^13^, and the self-made Nursing Satisfaction Rating Scale^14^. The Cronbach's a coefficient of the nursing satisfaction rating scale was 0.823, indicating good internal consistency. The cutoff score for the HAMA and HAMD scales was 14 points, with higher scores indicating more severe negative psychological changes. The Cronbach's α coefficient of the HAMA and HAMD scales was 0.895 and 0.876, respectively, indicating good internal consistency. The Nursing Satisfaction Rating Scale includes postoperative care and complications management, with a total score of 100 points. Higher scores indicate greater satisfaction among PPI-dependent GERD patients. We also recorded the incidence of postoperative complications, such as dysphagia, bloating, nausea, and infection, in both groups.

### Statistical analysis

Data analysis was performed using Statistical Product and Service Solutions (SPSS) 22.0 software (IBM, Armonk, NY, USA). Continuous data (postoperative gastrointestinal function recovery indicators of the two groups of PPI-dependent GERD patients after LF surgery) were presented as mean ± standard deviation (x̅±s), and differences were analyzed using the unpaired t-test. The incidence of complications (postoperative complications of the two groups of PPI-dependent GERD patients after LF surgery) was presented as a percentage, and differences were analyzed using the chi-square test. P < 0.05 was considered statistically significant.

## Results

### Comparison of recovery of gastrointestinal function after LF in PPI-dependent GERD patients between the two groups

Group A PPI-dependent GERD patients had significantly shorter time to first flatus, time to first feeding, time to first ambulation, and length of hospital stay after LF than group B (P < 0.05). See Table 1.

**Table 1:** Comparison of gastrointestinal function recovery after LF in PPI-dependent GERD patients between the two groups (x̅±*s*)

Group	Number of cases	First flatus time (d)	Time to first meal (d)	Time to first ambulation (d)	Length of stay (d)
Group A	49	1.72 ± 0.34	2.08 ± 0.51	1.33 ± 0.17	4.05 ± 0.65
Group B	49	1.89 ± 0.46	2.35 ± 0.68	1.49 ± 0.23	5.14 ± 0.72
χ^2^ value		2.080	2.223	3.915	7.865
P value		0.040	0.028	0.000	0.000

Comparison of pain severity at different time points before and after LF surgery between the two groups in patients with PPI-dependent GERD

**Figure 1:**
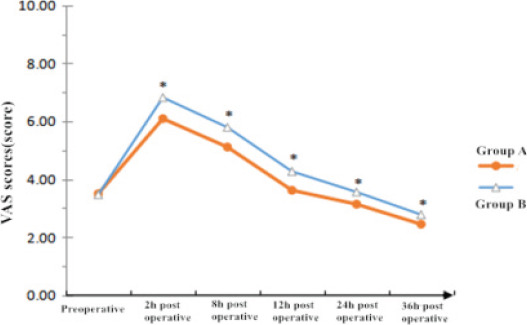
Comparison of VAS score at different time points before and after LF surgery in two groups PPI-dependent GERD patients

There was no significant difference in preoperative VAS scores of LF between the two groups of PPI-dependent GERD patients (P > 0.05); 2 h ∼ 36 h after operation, the VAS scores of both groups showed a tendency of first increase and then decrease, in which the VAS scores at each time point after LF surgery in group A were significantly lower than those in group B (F time = 10.259, Fig. F group between = 58.314, F time × group = 92.467, P < 0.05). See Figure 1.

Comparison of negative psychology before and after intervention between the two groups of PPI-dependent GERD patients

Before intervention, there was no significant difference in HAMA score and HAMD score between the two groups of PPI-dependent GERD patients (P > 0.05); after intervention, HAMA score and HAMD score in the two groups were significantly lower than those before intervention, and HAMA score and HAMD score in group A were lower than those in group B (P < 0.05). See Table 2.

**Table 2:** Comparison of negative psychology before and after intervention in two groups of PPI-dependent GERD patients (points, x̅ ± s)

Group	Number of cases	HAMA score		HAMD score	

		Pre-intervention	Post Intervention	Pre-intervention	Post Intervention
Group A	49	15.67 ± 1.23	6.01 ± 0.43	15.18 ± 1.95	6.42 ± 0.51
Group B	49	15.64 ± 1.41	10.54 ± 0.98	15.25 ± 1.82	12.07 ± 1.93
t-value		0.112	29.630	0.183	19.812
P-value		0.910	0.000	0.854	0.000

Comparison of nursing satisfaction after intervention between the two groups of PPI-dependent GERD patients

After implementation of different care regimens, patients with PPI-dependent GERD in group A had higher scores of various care satisfaction after nursing intervention than those in group B (P < 0.05). See Table 3. Comparison of the incidence of complications after LF surgery in patients with PPI-dependent GERD between the two groups

**Table 3:** Comparison of satisfaction with care after intervention between the two groups of PPI-dependent GERD patients (points, ± *s*)

Group	Number of cases	Basic nursing	Safe Care	Comfort Care	Social support	Psychological nursing
Group A	49	19.12 ± 0.23	19.15 ± 0.29	19.07 ± 0.28	18.97 ± 0.23	18.94 ± 0.24
Group B	49	18.47 ± 0.50	18.72 ± 0.34	18.29 ± 0.32	18.54 ± 0.37	18.64 ± 0.35
t-value		8.267	6.735	12.840	6.909	1.948
P-value		0.000	0.000	0.000	0.000	0.000

Group A The incidence of complications after LF surgery in PPI-dependent GERD patients was 12.24%, which was not significantly different from 16.33% in group B (P > 0.05). See Table 4.

**Table 4:** Comparison of incidence of complications after LF surgery in PPI-dependent GERD patients between the two groups [case (%)]

Group	Number of cases	Bloating	Nausea	Dysphagia	Infected	Overall complication rate
Group A	49	2 (4.08)	3 (6.12)	1 (2.04)	0 (0.00)	6 (12.24)
Group B	49	3 (6.12)	2 (4.08)	2 (4.08)	1 (2.04)	8 (16.33)
χ^2^-value						0.333
P-value						0.564

## Discussion

Laparoscopic fundoplication (LF) is a reliable surgical option for the clinical treatment of gastroesophageal reflux disease (GERD). It can effectively reduce acid exposure, improve esophageal motility and gastric emptying, and lower the risk of esophageal cancer precursors associated with severe GERD, while avoiding the side effects of long-term use of proton pump inhibitors (PPIs)^15^. Studies have found that comprehensive and effective perioperative nursing interventions can deepen GERD patients' understanding of LF surgery and have a positive impact on reducing the incidence of postoperative adverse events^16^. This study mainly investigates and analyzes the application effects of multi-perspective nursing based on the Enhanced Recovery After Surgery (ERAS) concept in LF for PPI-dependent GERD patients.

The results of this study showed that the first anal exhaust time, first meal time, first out-of-bed time, and hospital stay of Group A PPI-dependent GERD patients after LF surgery were significantly shorter than those of Group B. Additionally, the visual analogue scale (VAS) scores at different time points from 2 to 36 hours after surgery were significantly lower in Group A than in Group B, indicating that multi-perspective nursing based on the ERAS concept can effectively promote the recovery of gastrointestinal function and reduce the degree of postoperative pain response in PPI-dependent GERD patients compared to conventional nursing interventions. Although our VAS score showed no significant difference in preoperative pain levels between the two groups, Figure 1 in the manuscript reveals a baseline pain score of about 3.5. This apparent difference could be due to a number of factors. First, the subjective experience of pain may vary between individuals, and the VAS score is only an objective quantitative measure. Secondly, the pain before surgery may be related to the patient's disease condition and the treatment measures taken before. Patients with GERD often experience pain caused by acid reflux, which can be a major source of their preoperative pain. In addition, individual patient differences, lifestyle, and medication use may also play a role in preoperative pain scores.

While previous clinical nursing interventions using conventional nursing methods for PPI-dependent GERD patients undergoing LF surgery have achieved certain results, their preventive effect on postoperative complications is relatively limited^17^. Based on the ERAS concept, a multi-perspective care approach can optimize and improve perioperative care measures by analyzing common issues in perioperative care from the perspectives of patients, family members, and healthcare providers. In combination with the specific condition and surgical process of PPI-dependent GERD patients and the ERAS concept, the importance of gastrointestinal function recovery is emphasized, with the aim of promoting postoperative recovery and relieving pain^18^.

The results of this study show that after nursing intervention, patients with PPI-dependent GERD in Group A had lower HAMA and HAMD scores than those in Group B. Satisfaction scores for nursing care, including basic care, safety care, comfort care, social support, and psychological care, were significantly higher in Group A than in Group B. This indicates that compared to conventional nursing interventions based on the ERAS concept, the multi-perspective nursing interventions based on the ERAS concept can more effectively improve the negative psychological effects of patients with PPI-dependent GERD during the perioperative period and increase nursing satisfaction.

Multi-perspective nursing realizes multi-faceted and multi-angle perioperative care of patients from health status, physiological function, social function, emotional intelligence, and physical vitality, and provides personalized nursing interventions comprehensively. In addition to routine patient education related to PPI-dependent GERD, special attention is paid to the patient's psychological state, existing nursing problems, and special nursing needs of patients and their families.

The significant reduction in Visual Analogue Scale (VAS) pain scores among Group A patients at different time points after surgery underscores the positive impact of multi-perspective nursing on pain management. The multi-dimensional care provided in Group A may have collectively contributed to the reduction in pain responses by addressing not only the physiological aspects of pain but also psychological factors. Moreover, the lower Hamilton Anxiety Scale (HAMA) and Hamilton Depression Scale (HAMD) scores in Group A indicate that the multi-perspective nursing approach could effectively mitigate anxiety and depression during the perioperative period.

The higher nursing satisfaction scores in Group A are indicative of the enhanced patient experience resulting from multi-perspective care. The personalized attention and holistic support offered through this approach fostered better nurse-patient communication, instilled confidence in patients, and promoted a sense of emotional well-being. By addressing patients' psychological and social needs, the multi-perspective nursing interventions created a more patient-centered care environment.

In this study, Group A implemented a multi-perspective nursing strategy based on the ERAS concept, which comprehensively evaluated the patient's condition through the formation of a nursing intervention team. Through a multi-perspective approach, including medical, patient, and basic nursing care, psychological support care, etc., the ERAS concept nursing plan was implemented during the perioperative period of LF surgery to prevent and intervene in anxiety and depression in patients. Basic care, safety care, comfort care, social support, and psychological care were provided to achieve the goal of effectively improving the satisfaction of perioperative nursing intervention for LF surgery.

In this study, there was no significant difference in the incidence of complications after LF surgery in patients with PPI-dependent GERD in Group A and Group B. Specifically, the multi-perspective nursing interventions based on the ERAS concept can improve the cognitive level of patients with PPI-dependent GERD to some extent. By establishing good communication and psychological care, the multi-perspective nursing interventions are more feasible and practical compared to conventional nursing interventions during the perioperative period of LF surgery.

## Conclusion

Based on the comprehensive ERAS approach, multi-perspective care can effectively promote postoperative gastrointestinal function recovery, alleviate pain responses, improve psychological health status, and increase nursing satisfaction in PPI-dependent GERD patients after LF surgery. The limitations of this study are the small sample size and short intervention period, which require further follow-up investigations with increased sample sizes and extended observation time to evaluate the long-term effects of this model.
